# Influence of a Six-Week Swimming Training with Added Respiratory Dead Space on Respiratory Muscle Strength and Pulmonary Function in Recreational Swimmers

**DOI:** 10.3390/ijerph17165743

**Published:** 2020-08-08

**Authors:** Stefan Szczepan, Natalia Danek, Kamil Michalik, Zofia Wróblewska, Krystyna Zatoń

**Affiliations:** 1 Department of Swimming, Faculty of Physical Education, University School of Physical Education in Wroclaw, Ignacego Jana Paderewskiego 35, Swimming Pool, 51-612 Wroclaw, Poland; krystyna.zaton@awf.wroc.pl; 2 Department of Physiology and Biochemistry, Faculty of Physical Education, University School of Physical Education in Wroclaw, Ignacego Jana Paderewskiego 35, P-3 Building, 51-612 Wroclaw, Poland; natalia.danek@awf.wroc.pl (N.D.); kamil.michalik@awf.wroc.pl (K.M.); 3 Faculty of Pure and Applied Mathematics, Wroclaw University of Science and Technology, Zygmunta Janiszewskiego 14a, C-11 Building, 50-372 Wroclaw, Poland; zofia.wroblewska12@gmail.com

**Keywords:** swimming, added respiratory dead space, respiratory muscle strength, pulmonary function, respiratory variables

## Abstract

The avoidance of respiratory muscle fatigue and its repercussions may play an important role in swimmers’ health and physical performance. Thus, the aim of this study was to investigate whether a six-week moderate-intensity swimming intervention with added respiratory dead space (ARDS) resulted in any differences in respiratory muscle variables and pulmonary function in recreational swimmers. A sample of 22 individuals (recreational swimmers) were divided into an experimental (E) and a control (C) group, observed for maximal oxygen uptake (VO_2_max). The intervention involved 50 min of front crawl swimming performed at 60% VO_2_max twice weekly for six weeks. Added respiratory dead space was induced via tube breathing (1000 mL) in group E during each intervention session. Respiratory muscle strength variables and pulmonary and respiratory variables were measured before and after the intervention. The training did not increase the inspiratory or expiratory muscle strength or improve spirometric parameters in any group. Only in group E, maximal tidal volume increased by 6.3% (*p* = 0.01). The ARDS volume of 1000 mL with the diameter of 2.5 cm applied in moderate-intensity swimming training constituted too weak a stimulus to develop respiratory muscles and lung function measured in the spirometry test.

## 1. Introduction

Increased work of respiratory muscles can lead to their fatigue and a sense of dyspnoea, which, in turn, can impair the ability to perform physical exercise [1]. Respiratory muscle fatigue is defined as a loss in the capacity for developing force and/or velocity resulting from muscle activity under load, which reverses by rest [2]. It has been shown that the emerging respiratory muscle fatigue may be caused by the accumulation of metabolites in these muscles and sympathetic vasoconstriction in locomotor muscles as a result of the metabolic reflex of respiratory muscles [3]. This metaboreflex involves reduced blood flow in the extremities and thus decreased supply of oxygen (O_2_) to the respiratory muscles [4]. It is believed that the need for increased blood flow to the diaphragm is a potential reason for working muscle vasoconstriction, which can stimulate the development of locomotor muscle fatigue, reducing exercise tolerance [3,5].

The efficiency of the respiratory system depends not only on the amount of oxygen supplied by the cardiovascular system but also on the efficiency of removing the excess carbon dioxide (CO_2_) [6,7]. Increased minute ventilation (VE) during exercise allows for the adjustment of the partial pressure of CO_2_ in arterial blood (PaCO_2_), which can be measured noninvasively by establishing the end-tidal gas composition and its CO_2_ pressure (PetCO_2_) [8]. A high PaCO_2_ level implies an insufficient increase in VE during exercise and a limited ability to maximize exercise. This may be due to mechanical respiratory restrictions when reaching the upper limit of peak expiratory flow, such as insufficient respiratory muscle strength or reduced chemoreceptor reactivity. On the other hand, lower ventilation is also associated with reduced respiratory muscle work and can decrease blood flow in respiratory muscles, with a simultaneous increase (by ca. 10%) of extremity muscle blood flow. This mechanism can delay the appearance of fatigue [8]. This seems particularly important with reference to exercises in which VE pattern regulation, i.e., the adjustment of tidal volume (VT) and respiratory frequency (Rf), is associated with the rhythm of locomotor activities, e.g., swimming [9]. As respiratory muscle capacity is considered to be one of the many important factors determining exercise efficiency, it seems right to look for effective ways to increase it.

Respiratory muscle training (RMT) under normocapnic hyperpnoea conditions is applied to develop respiratory muscle strength and improve lung function [1,10]. Positive effects of this approach are based on several physiological adaptations, which include diaphragm hypertrophy, elevated nitric oxide concentration in the airways, change in the efficiency of muscle fibre contractions, improvement of the nervous control and economy of respiratory muscle work, delayed metabolic fatigue, reduced dyspnoea, and improved lung function [11,12,13]. These adaptations lead to decreases in the rating of perceived breathlessness or rating of perceived exertion. Moreover, the above mentioned attenuation of the metaboreflex phenomenon may result in the redirection of blood flow from locomotor muscles to respiratory muscles [1]. Studies lasting several weeks and using various types of devices to stimulate inspiratory resistance have already been conducted among trained swimmers [14,15]. However, similar research in recreational swimmers is still lacking. Knowledge of the training responses among this population should contribute to more effective training planning in order to counteract limiting the effort capacity of the respiratory system.

Other RMT methods that have been suggested involve breathing through a special mask [16] or tube breathing to increase the volume of the respiratory dead space [17]. As for the latter, the authors concluded that tube breathing was well tolerated by healthy individuals, did not cause desaturation or adverse events, and led to hypercapnia in most participants. In addition, it was speculated that a slight increase in PaCO_2_ during tube breathing might even provide a more intense training stimulus [17]. Previous research on exercise interventions including added respiratory dead space (ARDS) focused primarily on circulatory and respiratory responses to a single exercise session on a cycle ergometer [18] or on long-term training adaptations in physical performance during moderate-intensity continuous training in elite cyclists [19,20,21], high-intensity swim training in well-trained cohorts [22], or high-intensity interval training in amateur triathletes [23]. Our latest research examined the effects of swimming with ARDS on cardiorespiratory fitness and lipid metabolism among recreational swimmers [24]. While the cardiorespiratory response to ARDS is better understood, no studies to date have investigated the effects of a long-term intervention (six weeks) with ARDS on respiratory muscle variables and pulmonary function in recreational swimmers; this is therefore the subject of our present considerations. Increasing the distance that the air has to cover to reach the lungs raises airway resistance. The higher the gas flow rate, the higher the friction forces [25]. The compensatory mechanism (as in the case of raised respiratory minute ventilation) consists in increasing the tidal volume and decreasing the respiratory frequency. Poon [26] explains this in terms of so-called mechanical and respiratory optimization, as the body determines the value of ventilation that allows to bear the lowest possible cost of the respiratory muscles work in response to chemoreceptor pulsation. In this context, the aims of this study were to investigate whether there appeared any differences in respiratory muscle variables and pulmonary function after a six-week moderate-intensity swimming intervention with ARDS in recreational swimmers, as well as to determine if there were any performance advantages of applying a low-cost method that could safely induce ARDS. This information may be used by recreational swimmers to improve their pulmonary function, by coaches to support making decisions on enhancing the performance of developmental level and trained swimmers during the workout process and by untrained individuals to increase their pulmonary function and health. It was hypothesized that the ARDS intervention would bring about large improvements in respiratory muscle strength and beneficial changes in pulmonary variables. To our knowledge, this theory has not been empirically addressed yet. The rationale behind these postulates comes from the research on ARDS which has shown positive effects on selected cardiorespiratory variables.

## 2. Materials and Methods

### 2.1. Participants

The research involved 22 healthy and physically active people, including women (*n* = 11) and men (*n* = 11). Their physical activity was limited to swimming the average distance of 2 km twice a week with an intensity of 65–75% of maximum heart rate (HRmax). The participants were divided into 2 groups, the control group (C: 7 men, 4 women) and the experimental group (E: 4 men, 7 women) (Table 1). During the first visit, all participants’ body mass (kg) and height (m) were measured by using WPT 200 medical scales (RADWAG, Radom, Poland). The groups were compared in terms of the somatic parameters, i.e., age (*p* = 0.72), body height (*p* = 0.50), body mass (*p* = 0.65), and maximal oxygen uptake (VO_2_max) (*p* = 0.65), and the Wilcoxon nonparametric test was applied in the assessment (alpha error: 0.05). This generated comparable groups with an objective baseline level of somatic build.

The individuals’ assignment to the study groups was based on VO_2_max values measured during a progressive test performed in accordance with the protocol by Michalik et al. [27] on an Excalibur Sport cycle ergometer (Lode BV, Groningen, the Netherlands) 3 days prior to the ARDS intervention. The VO_2_max values were arranged from highest to lowest. The study participants were ascribed sequential numbers in accordance with their VO_2_max results. The individuals with odd numbers were assigned to group E and those with even numbers to group C. Before entering the experiment, all swimmers provided their written consent to participate in the study; they could withdraw at any time. The experiment was approved by the University Research Ethics Committee (#14/2017) and carried out in accordance with the standards of the Declaration of Helsinki.

### 2.2. Design and Procedures

Added respiratory dead space intervention protocol.

A week before the start of the tests, a familiarization session was held to adapt the participants to the study protocol with ARDS, as none of them had previously used this method. The familiarization session involved a 1000-mL low-intensity front crawl swimming in a 25-m indoor swimming pool, with breathing through an ARDS device.

The participants in group E took part in a 6-week ARDS training. During the 6 weeks, they completed a total of 12 swimming sessions with ARDS. The ARDS intervention was limited to 2 swimming sessions per week. During each 50-min session, the individuals were front crawling. The interval between sessions was 72 h. During each swimming session, the participants undertook constant, moderate-intensity physical effort of aerobic character. The effort intensity was individually determined on the basis of the heart rate (HR) achieved at 60% VO_2_max in the progressive test, corresponding to individual HR values in the range of 125–140 beats min^–1^. While swimming, the participants monitored their HR with an RS400 sports watch (Polar Electro, Kempele, Finland). Intensity below the lactate threshold was chosen because it was suitable for long-term effort of untrained individuals involved in the experiment.

**Figure 1:**
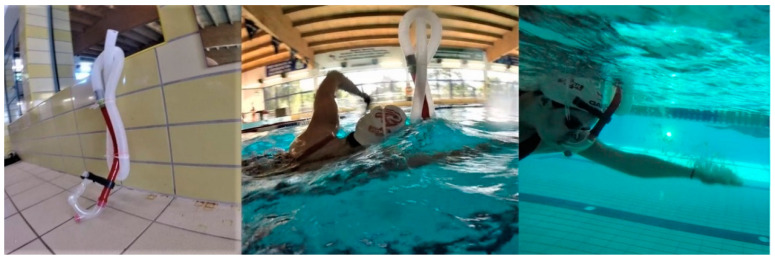
An instrument increasing added respiratory dead space: a custom added respiratory dead space (ARDS) apparatus consisting of a polypropylene centre-mount swimming snorkel with a mouthpiece (Speedo International Ltd., Nottingham, UK) integrated with 2.5-cm diameter ribbed tubing to provide 1000 mL of dead space.

Group E swam with a custom ARDS apparatus consisting of a polypropylene centre-mount swimming snorkel with a mouthpiece (Speedo International Ltd., Nottingham, UK) integrated with 2.5-cm diameter ribbed tubing to provide ARDS of 1000 mL (Figure 1). Dead space volume (1000 mL) was identical for each participant and measured by filling the snorkel with water and then transferring the volume to a graduated cylinder, as described by Szczepan et al. [24]. The snorkel was sufficiently rigid to maintain a constant volume when swimming.

The swimmers in group C took part in the same training but without ARDS intervention. In group C, no additional respiratory changes were introduced; the group applied a standard breathing pattern for the front crawl technique.

All sessions took place in a 25-m swimming pool, under uniform conditions (water temperature: 27 °C, air temperature: 28 °C, relative humidity: 60%, lighting: 600 lx). Throughout the experimental period, the individuals from both groups led a lifestyle and maintained a diet normal for people of that age and did not participate in any additional training. The participants’ diet was not controlled.

### 2.3. Independent Variable Measurements

Respiratory muscle, pulmonary function, and cardiorespiratory tests were administered 3 days before and after the intervention with ARDS to assess changes in respiratory muscle strength and pulmonary function between the pre- and postintervention status. Both testing series were performed in the same controlled conditions (temperature: 24 °C, relative humidity: 50%) in a climate-controlled exercise laboratory (PN-EN ISO 9001:2009 certified). The measurements were taken by a laboratory worker with a device calibrated before each trial.

#### 2.3.1. Respiratory Muscle Strength Variable Measurements

Inspiratory muscle strength (maximal inspiratory pressure [PImax] [cm H_2_O]) and expiratory muscle strength (maximal expiratory pressure [PEmax] [cm H_2_O]) were measured in a test using a Micro RPM respiratory pressure meter (CareFusion, San Diego, CA, USA). To assess PImax, the tested person, in a standing position, performed a maximum inspiration from the level of a maximum expiration. Then, to evaluate PEmax, the individual exhaled starting from the maximum inspiration level. In both cases, a special stopper was fitted. The PImax and PEmax tests were conducted at rest [28]. Each participant took 2 trials for maximum inspiration and maximum expiration each, and the higher values were selected for further analysis.

#### 2.3.2. Pulmonary Variable Measurements

Pulmonary function was measured by spirometry as a functional examination of the respiratory system. Spirometry was performed by using a Quark b^2^ ergospirometer (Cosmed, Milan, Italy). It involved an inspiration with a maximum volume preceded by 2–3 quiet breaths and ended with an intense exhalation with a maximum airflow, resulting in a minimum volume of residual air. In the course of the respiratory test, the following parameters were recorded: forced vital capacity (FVC) [L], forced expiratory volume in 1 s (FEV_1_) [L], peak expiratory flow (PEF) [L s^–1^], and peak inspiratory flow (PIF) [L s^–1^]. Each participant took 2 trials, and the one with higher FEV_1_ value was selected for further analysis.

#### 2.3.3. Respiratory Variable Measurements

An incremental exercise test on a cycle ergometer was applied to assess VO_2_max [mL kg^–1^ min^–1^], VE [L min^–1^], Rf [breaths min^–1^], VT [L breath^–1^] and other respiratory parameters: Total duration of the inspiratory cycle (Ti) [s], total duration of the expiratory cycle (Te) [s], total duration of the respiratory cycle (Ttot) [s], ratio of mean inspiratory time to the total time of the respiratory cycle (Ti/Ttot) [%], PetCO_2_ [mm Hg]. Heart rate [beats min^–1^] was also continuously measured with a noninvasive HR monitor (S810, Polar Electro, Kempele, Finland).

The incremental exercise test was administered 3 days before the training intervention. Gas exchange was evaluated breath-by-breath by using a metabolic cart (Quark b^2^, Cosmed, Italy). The device was calibrated with a reference gas mixture of CO_2_ (5%), O_2_ (16%), and N_2_ (79%). Pulmonary function assessment began 2 min prior to the test start and continued 5 min after the test conclusion, with data averaged over 30-s intervals. VO_2_ was measured and VO_2_max was automatically indicated. VO_2_max was defined as the highest 30-s average at which relative VO_2_ values plateaued (<1.35 mL kg^−1^ min^−1^) despite an increase in workload or 2 of the following criteria: (a) respiratory exchange ratio > 1.10; (b) attainment of HRmax (within 10 beats min^–1^ of age-predicted maximum [220-age]); (c) voluntary exhaustion. Primary cardiorespiratory outcome measures of Rf [breaths∙min^–1^], VT [L breath^–1^], VE [L min^–1^] were determined at 4 workloads (50, 100, 150, 200 W) and at maximal power (max). The outcome measures of Ti [s], Te [s], Ttot [s], Ti/Ttot [%], PetCO_2_ [mm Hg] were determined at 4 workloads (50, 100, 150, 200 W).

### 2.4. Statistical Analysis

The quantitative investigation planning involved a 4-dimensional approach (alpha, power, sample size, and effect size) and followed the accepted methodology [29].

Data are presented as means ± standard deviations, the difference (Δ) between pre- and postintervention values, and the standard deviation for the difference. In addition, parameter changes (increase or decrease) are expressed as a dimensionless ratio of two quantities (%). Significance was set at an alpha level of 0.05 for all statistical procedures, with *p* values provided for all results.

The distribution of the data set was screened for normality by using the Kolmogorov–Smirnov test. The homogeneity of variances was checked with the Levene’s test. Respiratory muscle strength variables (PImax, PEmax), pulmonary variables (FVC, FEV_1_, PEF, PIF), and respiratory variables (Rf, VT) for each workload (50, 100, 150, 200 W, max), and Ti, Te, Ttot, Ti/Ttot, PetCO_2_ were compared with the use of one-way ANOVA with repeated measures (measurement × group) and Tukey’s honest significant difference (HSD) test for pairwise posthoc comparisons. The VE and VO_2_max variables values derived from Szczepan et al. [25].

Furthermore, effect sizes for ANOVA were calculated by using partial eta squared ($\eta_{p}^{2}$). Effect sizes were interpreted as small (0.02), moderate (0.13), or large (≥0.26) [30,31].

The sample size was estimated with a stand-alone power analysis program for statistical tests (G*Power 3.1.9.2, Kiel University, Kiel, Germany) [32] with a small effect size of f2 = 0.29. With the assumption of an alpha error of 0.05 and power of (1-β) 0.80, the required total sample size was estimated to be 26 participants in total. However, owing to the length and commitment of the intervention, we were able to include only 22 individuals in the final analysis.

All calculations of the analysed variables were performed with the IBM SPSS Statistics version 26 software package (IBM, Inc., Chicago, IL, USA).

## 3. Results

Pre- and postintervention respiratory muscle strength, pulmonary function, and respiratory outcomes for within-group comparisons are presented in Table 1, Table 2 and Table 3. Between-group comparisons are provided in the text only.

No between- or within-group differences were observed after the intervention for respiratory muscle strength variables (PImax, PEmax) or pulmonary/spirometry variables (FVC, FEV_1_, PEF, PIF) (Table 2).

Among respiratory variables (Rf, VT, VE), the difference analysis revealed changes only within the experimental group for the VT variable at 100 W workload (decrease by 13.7%; *p* = 0.03; $\eta_{p}^{2}$ = 0.39) and at maximum workload (increase by 6.3%; *p* = 0.01; $\eta_{p}^{2}$ = 0.52) (Table 3). Pre- and postintervention between-group comparisons (control group vs. experimental group) did not indicate any changes.

For the other respiratory variables (Ti, Te, Ttot, Ti/Ttot, PetCO_2_), the difference analysis showed changes within the control group for the Ti/Ttot variable at 150 W workload (decrease by 2.1%; *p* = 0.01; $\eta_{p}^{2}$ = 0.46) and at 200 W workload (decrease by 2.0%; *p* = 0.04; $\eta_{p}^{2}$ = 0.36). Differences were also observed within the control group for PetCO_2_ at 200 W workload (decrease by 2.7%; *p* = 0.02; $\eta_{p}^{2}$ = 0.44). Changes were recorded within the experimental group for the Ti variable at 100 W workload (decrease by 16.7%; *p* = 0.01; $\eta_{p}^{2}$ = 0.52), Ttot at 100 W workload (decrease by 11.5%; *p* = 0.02; $\eta_{p}^{2}$ = 0.45), Ti/Ttot at 100 W workload (decrease by 4.4%; *p* = 0.04; $\eta_{p}^{2}$ = 0.35), and PetCO_2_ at 100 W workload (decrease by 2.6%; *p* = 0.01; $\eta_{p}^{2}$ = 0.47) and at 150 W workload (decrease by 5.6%; *p* = 0.04; $\eta_{p}^{2}$= 0.35) (Table 4).

In turn, between-group comparisons showed pre-intervention differences for the Te variable at 100 W workload (Δ = 0.41; *p* = 0.03; $\eta_{p}^{2}$ = 0.21) and 150 W workload (Δ = 0.34; *p* = 0.02; $\eta_{p}^{2}$ = 0.23) and for the Ti/Ttot variable at 150 W workload (Δ = −2.0; *p* = 0.03; $\eta_{p}^{2}$ = 0.22) and 200 W workload (Δ = −3.0; *p* = 0.01; $\eta_{p}^{2}$ = 0.31).

## 4. Discussion

The main finding of the study is that a six-week ARDS intervention of moderate intensity (HR: 125–140 beats min^–1^) did not significantly change respiratory muscle strength (PImax, PEmax) or spirometric parameters (FVC, FEV_1_, PEF, PIF), which did not confirm the assumed hypothesis. Interestingly, only in group E, maximal tidal volume increased by 5.5%.

Research on the use of ARDS to improve cardiopulmonary capacity in different exercise regimes and intensities is common [19,20,21,22,23,33]. However, to the best of our knowledge, this is the first study to analyse the effects of ARDS application during moderate-intensity swimming in recreational swimmers on changes in lung functional parameters and respiratory muscle strength. Studies suggest that swimming is an activity extremely demanding for inspiratory muscles since immersion in water forces swimmers to expand the chest wall under higher pressure and to increase both VT and the speed of muscle contraction, which can lead to premature appearance of fatigue symptoms [9]. We assumed that the use of ARDS during swimming would be a stronger stimulus for the development of respiratory muscle strength and lung function measured by spirometry.

In earlier research, the use of ARDS led to CO_2_ accumulation above the physiological norm, triggering changes in the respiratory system, increasing VE by raising Rf and VT, and causing faster respiratory muscle fatigue [17,34]. This means that breathing with additional difficulty due to the increased respiratory resistance requires the involvement of greater respiratory muscle strength, which reduces lung susceptibility and, consequently, increases respiratory muscle endurance [35,36]. RMT and its variations employing high ventilation rates and generating high respiratory pressure improved PImax and VO_2_max [37]. Resistance RMT (RRMT), involving application of efforts at increased respiratory resistance, led to improvements in PImax, PEmax, and VT [11]. Apnoea training, raising tolerance to hypoxaemia regardless of the genetic factor or muscle buffer capacity, shortened the time of 400-m front crawl [38]. In addition, Karaula et al. [39] revealed that the application of the hypercapnic-hypoxic respiratory pattern significantly improved the strength of inspiratory and expiratory muscles, by 14.9% and 1.9%, respectively, compared with the control group swimmers. Similarly, McEntire et al. [40] pointed out that the use of a device raising respiratory resistance and regular breathing exercises increased respiratory muscle strength. The results of our research are contrary to many experiments in which different RMT stimuli were used. Among the factors that may explain the lack of changes in spirometric parameters (FVC, FEV_1_, PEF, PIF) and respiratory muscle strength parameters (PImax, PEmax) observed in our study, there is the application of too low a swimming intensity with 2.5-cm diameter ARDS, which did not generate sufficiently high inspiratory pressure. Enright et al. [41] suggest that most gains in inspiratory muscle strength occur at an intensity of PImax. We are unable to determine what inspiratory pressure was generated by the participants during the swimming sessions in the presented experiment. Therefore, further studies could be undertaken to clarify this issue.

High PaCO_2_ (provoked by ARDS) irritates cardiovascular chemoreceptors and increases VE, mainly by raising VT [42]. Regular hypercapnia can also modify the reactivity of chemoreceptive areas and thus change the respiratory pattern [20]. McParland et al. [43] report that the application of ARDS (970 mL) increased VT, as opposed to Rf. Our results do not confirm these observations, indicating lack of differences in maximal and submaximal VE. However, during work with 100 W intensity, the progressive test in group E showed a decrease in VT without Rf changes. This may indicate an improvement in work economy as a result of applying similar intensity in training. This is in line with the findings provided by Michalik et al. [44], who implied an improvement in exercise economy in a progressive test with the intensity that had been used in the training process. After a six-week swimming training with ARDS, group E presented a decrease in VT accompanied by lower Ttot, Ti, and Ti/Ttot values. No such changes occurred in the control group. According to Buchler et al. [45], lowering Ti/Ttot increases blood flow in the diaphragm to provide more oxygen to the inspiratory muscles, which may also explain the increase in the maximum VT value as a result of a lower physiological cost of respiratory muscle work. The raised oxygen supply to the diaphragm can delay the occurrence of fatigue and thus improve exercise tolerance [3]. Unfortunately, we did not test blood PaCO_2_ or the respiratory pattern during swimming sessions, and this knowledge could help interpret the results. It seems that even if hypercapnia was induced, the ventilation response was too weak a stimulus to induce long-term adaptation.

In group E, the value of PetCO_2_ at an intensity of 100 and 150 W decreased. Similar results were observed in group C but at an intensity of 100 W. Changes in PetCO_2_ during the progressive test may indicate a change in muscle metabolism and in chemoreceptor sensitivity to CO_2_ and H^+^ modifications [8]. In the previous study [24], we showed that CO_2_ excretion did not change as a result of ARDS training. Thus, the lower PetCO_2_ in the present study is associated with a more efficient CO_2_ elimination by the lungs, as evidenced by the synergistic effect of the VE components mentioned above (VT, Rf). However, this conclusion requires further research and detailed verification in subsequent studies including measurements during training sessions.

Nevertheless, the presented results should be interpreted with caution. The study limitations include the small size of both groups. In addition, the progressive test was carried out in laboratory conditions on a cycle ergometer and therefore did not take into account the horizontal position of the body in water. Field tests similar to the training sessions will be a more accurate way to determine the aerobic capacity of swimmers. This approach can provide more sensitive data to enable a better direction of training, consequently facilitating improved performance. We applied the ARDS volume of 1000 mL and the tube diameter of 2.5 cm, as tested in previous studies, but these parameters were not adjusted to the individual vital capacity of the participants. The absence of significant changes in most of the measured characteristics may suggest that either the exercise stimulus was too small (low intensity) or the application time was too short. It is advisable to consider a higher intensity of training, e.g., second ventilatory (anaerobic) threshold, which would increase VE and respiratory muscle involvement. Dunham and Harms [28] proved that the stimulus to induce respiratory muscle adaptation required high-intensity work, as in the case of high-intensity interval training that they applied. Further changes to the ARDS training protocol, regarding frequency (number of training units per week) and volume (number of intervention weeks), may also cause other body reactions. In addition, the design of the device to increase the dead space can be altered, e.g., by reducing the tube diameter, in order to induce higher respiratory resistance and monitor the respiratory gas parameters in real time to determine changes in, among others, PetCO_2_. Future studies should take these limitations into account.

## 5. Conclusions

Summing up, this study has shown for the first time that a six-week moderate-intensity training with the application of 1000-mL ARDS among recreational swimmers does not cause changes in respiratory muscle strength variables and pulmonary variables.

## Figures and Tables

**Table 1:** Participants’ characteristics (mean ± standard deviation).

Variables	E	C
Age (years)	24.3 ± 2.7	24.0 ± 3.3
Body height (m)	1.7 ± 0.1	1.7 ± 0.1
Body mass (kg)	70.0 ± 13.1	72.3 ± 10.1
VO_2_max (mL kg^–1^ min^–1^)	45.6 ± 7.5	47.1 ± 8.9

VO_2_max—maximal oxygen uptake.

**Table 2:** Pre- and postintervention within-group comparisons (PImax, PEmax, FVC, FEV_1_, PEF, PIF).

Control Group							
Variables	Pre-Intervention	Post-Intervention	Δ (Post-Pre)	± of Δ (Post-Pre)	% Difference	*p* Value	$\eta_{p}^{2}$
PImax [cm H_2_O]	127.6 ± 38.1	124.1 ± 36.2	−3.5	52.6	−2.7	0.47	0.05
PEmax [cm H_2_O]	162.6 ± 33.0	166.5 ± 32.5	3.8	46.3	2.3	0.46	0.06
FVC [L]	6.6 ± 1.6	6.5 ± 1.6	−0.1	2.2	−1.8	0.68	0.02
FEV_1_ [L]	4.8 ± 0.9	4.9 ± 1.0	0.1	1.4	1.9	0.74	0.01
PEF [L s^–1^]	8.9 ± 2.2	8.9 ± 1.9	0.0	2.9	0.3	0.89	0.01
PIF [L s^–1^]	2.6 ± 0.9	2.2 ± 1.0	−0.5	1.4	−18.3	0.10	0.24
**Experimental Group**							
**Variables**	**Pre-Intervention**	**Post-Intervention**	**Δ (Post-Pre)**	**± of Δ (Post-Pre)**	**% Difference**	***p*** **Value**	**$\eta_{p}^{2}$**
PImax [cm H_2_O]	122.9 ± 40.7	131.2 ± 26.4	8.3	48.5	6.7	0.47	0.05
PEmax [cm H_2_O]	136.1 ± 52.8	156.6 ± 49.0	20.4	72.0	15.0	0.21	0.01
FVC [L]	6.0 ± 1.2	6.1 ± 1.6	0.1	2.0	1.5	0.80	≥0.00
FEV_1_ [L]	4.9 ± 0.9	4.6 ± 0.9	−0.3	1.2	−5.4	0.22	0.15
PEF [L s^–1^]	8.2 ± 2.2	7.9 ± 2.2	−0.3	3.1	−3.6	0.58	0.03
PIF [L s^–1^]	1.9 ± 1.0	2.6 ± 1.8	0.7	2.1	34.7	0.26	0.13

Data presented as mean ± standard deviation. Δ and % difference with respect to preintervention status. Positive Δ indicates an increase in variables. Positive % indicates an increase in variables. ± of Δ (post-pre)—standard deviation for the difference. PImax—maximal inspiratory pressure, PEmax—maximal expiratory pressure, FVC—forced vital capacity; FEV_1_—forced expiratory volume in 1 s; PEF—peak expiratory flow, PIF—peak inspiratory flow.

**Table 3:** Pre- and postintervention within-group comparisons (Rf, VT, VE, VO_2_max).

Control Group								
Variables	Power [W]	Pre-Intervention	Post-Intervention	Δ (Post-Pre)	± of Δ (Post-Pre)	% Difference	*p* Value	$\eta_{p}^{2}$
Rf [breaths min^–1^]	50	20.4 ± 2.8	20.4 ± 5.5	0.0	6.2	−0.1	0.99	≥0.00
	100	23.1 ± 4.7	22.6 ± 4.0	−0.5	6.1	−2.0	0.68	0.02
	150	25.3 ± 4.1	25.9 ± 4.5	0.6	6.1	2.4	0.61	0.03
	200	29.5 ± 7.9	31.3 ± 6.6	1.8	10.2	6.0	0.22	0.15
	Max	47.8 ± 10.3	47.1 ± 7.6	−0.7	12.8	−1.4	0.72	0.01
VT [L breath^–1^]	50	1.4 ± 0.2	1.4 ± 0.3	0.1	0.4	3.6	0.65	0.02
	100	1.7 ± 2.0	1.7 ± 0.2	0.0	2.0	−1.8	0.65	0.02
	150	2.0 ± 0.3	2.1 ± 0.3	0.1	0.4	4.0	0.32	0.01
	200	2.4 ± 0.4	2.4 ± 0.3	0.0	0.5	−0.8	0.85	0.01
	Max	2.6 ± 0.6	2.6 ± 0.5	0.0	0.8	−0.8	0.82	≥0.00
VE [L min^–1^]	50	28.8 ± 4.8	28.2 ± 4.2	−0.6	6.4	−2.1	0.99	0.01
	100	39.0 ± 4.1	37.4 ± 2.6	−1.6	4.9	–4.1	0.80	0.11
	150	51.9 ± 5.3	53.4 ± 4.3	1.5	6.8	2.9	0.89	0.07
	200	71.5 ± 12.3	72.4 ± 7.0	0.9	14.2	1.3	0.99	0.01
	Max	132.3 ± 35.1	135.8 ± 39.8	3.5	53.1	2.6	0.93	0.03
VO_2_max [mL kg^–1^ min^–1^]	Max	47.1 ± 8.9	47.6 ± 10.2	0.5	13.5	1.1	0.97	0.05
**Experimental Group**								
**Variables**	**Power [W]**	**Pre-Intervention**	**Post-Intervention**	**Δ (Post-Pre)**	**± of Δ (Post-Pre)**	**% Difference**	***p*** **Value**	**$\eta_{p}^{2}$**
Rf [breaths min^–1^]	50	18.2 ± 5.1	18.5 ± 5.9	0.3	7.8	1.4	0.87	≥0.00
	100	19.5 ± 6.3	20.9 ± 4.8	1.4	8.0	7.1	0.36	0.09
	150	22.1 ± 7.0	23.3 ± 5.3	1.2	8.7	5.5	0.52	0.04
	200	28.8 ± 8.7	27.9 ± 8.0	−0.9	11.8	−3.2	0.58	0.03
	Max	41.8 ± 6.2	40.5 ± 7.9	−1.2	10.1	−2.9	0.60	0.03
VT [L breath^–1^]	50	1.6 ± 0.5	1.4 ± 0.5	−0.1	0.7	−8.3	0.15	0.2
	100	2.1 ± 0.7	1.8 ± 0.4	−0.3	0.8	−13.7	0.03 *	0.39
	150	2.3 ± 0.6	2.2 ± 0.4	−0.1	0.7	−5.7	0.34	0.09
	200	2.4 ± 0.5	2.5 ± 0.5	0.1	0.7	4.6	0.40	0.07
	Max	2.5 ± 0.6	2.7 ± 0.6	0.2	0.8	6.3	0.01 *	0.52
VE [L min^–1^]	50	26.9 ± 6.0	24.6 ± 3.3	−2.3	6.8	−8.6	0.63	0.11
	100	36.9 ± 5.0	35.6 ± 2.1	−1.3	5.4	−3.5	0.84	0.05
	150	47.6 ± 6.2	48.7 ± 3.8	1.1	7.3	2.3	0.95	0.02
	200	66.4 ± 12.0	66.8 ± 7.4	0.4	14.1	0.6	1.00	0.01
	Max	121.5 ± 39.5	124.8 ± 37.1	3.3	54.2	2.7	0.94	0.04
VO_2_max [mL kg^–1^ min^–1^]	Max	45.6 ± 7.5	46.7 ± 8.3	1.1	11.2	2.4	0.80	0.15

Data presented as mean ± standard deviation. * Significant difference at *p* < 0.05 vs. preintervention value. Δ and % difference with respect to preintervention status. Positive Δ indicates an increase in variables. Positive % indicates an increase in variables. ± of Δ (post-pre)—standard deviation for the difference. Rf—respiratory frequency, VT—tidal volume, VE—respiratory minute ventilation, VO_2_max—maximal oxygen uptake. The VE and VO_2_max variables values derived from Szczepan et al. [25].

**Table 4:** Pre- and postintervention within-group comparisons (Ti, Te, Ttot, Ti/Ttot, PetCO_2_).

Control Group								
Variables	Power [W]	Pre-Intervention	Post-Intervention	Δ (Post-Pre)	± of Δ (Post-Pre)	% Difference	*p* Value	$\eta_{p}^{2}$
Ti [s]	50	1.4 ± 0.2	1.4 ± 0.4	0.1	0.4	4.4	0.62	0.03
	100	1.3 ± 0.3	1.3 ± 0.2	0.0	0.4	−0.8	0.92	≥0.00
	150	1.2 ± 0.2	1.1 ± 0.2	−0.1	0.3	−6.0	0.20	0.18
	200	1.1 ± 0.3	1.0 ± 0.2	−0.1	0.4	−12.0	0.11	0.24
Te [s]	50	1.6 ± 0.3	1.7 ± 0.4	0.1	0.5	4.9	0.52	0.04
	100	1.4 ± 0.3	1.5 ± 0.2	0.0	0.4	2.1	0.68	0.02
	150	1.3 ± 0.2	1.3 ± 0.2	0.0	0.3	0.8	0.79	0.01
	200	1.1 ± 0.3	1.0 ± 0.2	−0.1	0.3	−4.6	0.32	0.01
Ttot [s]	50	3.0 ± 0.4	3.1 ± 0.8	0.1	0.8	4.7	0.56	0.04
	100	2.7 ± 0.6	2.7 ± 0.4	0.0	0.7	1.1	0.87	≥0.00
	150	2.4 ± 0.4	2.4 ± 0.4	−0.1	0.6	−2.5	0.56	0.03
	200	2.2 ± 0.6	2.0 ± 0.4	−0.2	0.7	−8.3	0.16	0.17
Ti/Ttot [%]	50	45.0 ± 3.0	44.0 ± 3.0	−1.0	4.2	−2.2	0.51	0.05
	100	46.0 ± 3.0	46.0 ± 2.0	0.0	3.6	0.0	0.36	0.08
	150	47.0 ± 2.0	46.0 ± 2.0	−1.0	2.8	−2.1	0.01 *	0.46
	200	49.0 ± 2.0	48.0 ± 2.0	−1.0	2.8	−2.0	0.04 *	0.36
PetCO_2_ [mm Hg]	50	38.0 ± 1.8	37.6 ± 3.2	−0.4	3.6	−1.0	0.63	0.02
	100	39.5 ± 2.4	40.4 ± 3.0	0.9	3.8	2.3	0.15	0.20
	150	40.9 ± 2.7	39.9 ± 2.8	−1.0	3.9	−2.4	0.18	0.17
	200	40.3 ± 2.8	39.2 ± 2.7	−1.1	3.9	−2.7	0.02 *	0.44
**Experimental Group**								
**Variables**	**Power [W]**	**Pre-Intervention**	**Post-Intervention**	**Δ (Post-Pre)**	**± of Δ (Post-Pre)**	**% Difference**	***p*** **Value**	**$\eta_{p}^{2}$**
Ti [s]	50	1.5 ± 0.4	1.5 ± 0.5	0.0	0.6	−0.7	0.94	≥0.00
	100	1.6 ± 0.5	1.3 ± 0.3	−0.3	0.6	−16.7	0.01 *	0.52
	150	1.3 ± 0.4	1.2 ± 0.3	−0.1	0.5	−8.2	0.36	0.08
	200	1.0 ± 0.2	1.1 ± 0.2	0.0	0.3	2.9	0.51	0.04
Te [s]	50	2.0 ± 0.6	2.1 ± 0.7	0.0	0.9	1.5	0.81	≥0.00
	100	1.8 ± 0.5	1.7 ± 0.4	−0.1	0.7	−7.6	0.12	0.23
	150	1.6 ± 0.4	1.4 ± 1.4	−0.2	1.5	−10.0	0.21	0.16
	200	1.2 ± 0.3	1.2 ± 0.4	0.0	0.5	0.8	0.84	≥0.00
Ttot [s]	50	3.5 ± 1.0	3.6 ± 1.1	0.0	1.5	0.6	0.93	≥0.00
	100	3.4 ± 1.0	3.0 ± 0.7	−0.4	1.2	−11.5	0.02 *	0.45
	150	2.9 ± 0.8	2.7 ± 0.6	−0.3	1.0	−8.8	0.27	0.12
	200	2.3 ± 0.5	2.3 ± 0.6	0.0	0.7	1.8	0.71	0.02
Ti/Ttot [%]	50	42.0 ± 3.0	42.0 ± 2.0	0.0	3.6	0.0	0.43	0.06
	100	45.0 ± 2.0	43.0 ± 2.0	−2.0	2.8	−4.4	0.04 *	0.35
	150	45.0 ± 1.0	45.0 ± 2.0	0.0	2.2	0.0	0.38	0.08
	200	46.0 ± 2.0	46.0 ± 2.0	0.0	2.8	0.0	0.45	0.06
PetCO_2_ [mm Hg]	50	39.6 ± 3.1	38.7 ± 2.8	−0.9	4.2	−2.3	0.09	0.27
	100	41.5 ± 2.8	40.5 ± 2.5	−1.1	3.8	−2.6	0.01 *	0.47
	150	43.5 ± 4.1	41.0 ± 2.6	−2.5	4.9	−5.6	0.04 *	0.35
	200	41.1 ± 3.7	40.1 ± 3.6	−1.0	5.2	−2.4	0.22	0.15

Data presented as mean ± standard deviation. * Significant difference at *p* < 0.05 vs. preintervention value. Δ and % difference with respect to preintervention status. Positive Δ indicates an increase in variables. Positive % indicates an increase in variables. ± of Δ (post-pre)—standard deviation for the difference. Ti—total duration of the inspiratory cycle, Te—total duration of the expiratory cycle, Ttot —total duration of the respiratory cycle, Ti/Ttot—ratio of mean inspiratory time to the total time of the respiratory cycle, PetCO_2_—end-tidal partial pressure of carbon dioxide.
